# Novel Usefulness of M2BPGi for Predicting Severity and Clinical Outcomes in Hospitalized COVID-19 Patients

**DOI:** 10.3390/diagnostics15070937

**Published:** 2025-04-06

**Authors:** Mikyoung Park, Mina Hur, Hanah Kim, Chae Hoon Lee, Jong Ho Lee, Hyung Woo Kim, Minjeong Nam, Seungho Lee

**Affiliations:** 1 Department of Laboratory Medicine, Ajou University School of Medicine, Suwon 16499, Republic of Korea; mikyoung.pak@gmail.com; 2 Department of Laboratory Medicine, Konkuk University School of Medicine, Seoul 05030, Republic of Korea; md.hkim@gmail.com; 3 Department of Laboratory Medicine, Yeongnam University College of Medicine, Daegu 42415, Republic of Korea; chlee@med.yu.ac.kr (C.H.L.); ae4207@naver.com (J.H.L.); 4 Department of Laboratory Medicine, CHA Gumi Medical Center, CHA University, Gumi 39295, Republic of Korea; stevek1@naver.com; 5 Department of Laboratory Medicine, Korea University College of Medicine, Seoul 02841, Republic of Korea; mjnam0906@korea.ac.kr; 6 Department of Preventive Medicine, College of Medicine, Dong-A University, Busan 49201, Republic of Korea; lgydr1@gmail.com

**Keywords:** M2BPGi, COVID-19, severity, clinical outcome, SOFA, NEWS2

## Abstract

**Background/Objectives:** Mac-2 binding protein glycosylation isomer (M2BPGi) is a novel biomarker for liver fibrosis, and its prognostic role has never been explored in coronavirus disease 2019 (COVID-19). We compared the M2BPGi level simultaneously with age, severe/critical disease, the sequential organ failure assessment (SOFA) score, and the National Early Warning Score 2 (NEWS2) in a total of 53 hospitalized patients with COVID-19 (mild/moderate [*n* = 15] and severe/critical [*n* = 38]). **Methods:** M2BPGi levels were measured using the HISCL M2BPGi assay (Sysmex, Kobe, Japan) in an HISCL-5000 analyzer (Sysmex), and clinical outcomes were analyzed according to M2BPGi and the clinical variables, using the receiver operating characteristic (ROC) curve, Kaplan–Meier survival, and Cox proportional hazards regression analyses. **Results:** M2BPGi levels differed significantly according to disease severity, 30-day mortality, and 60-day mortality (*p* = 0.045, 0.011, and 0.002, respectively). In the ROC curve analysis, the M2BPGi, age, SOFA score, and NEWS2, except for severe/critical disease, significantly predicted clinical outcomes (all *p* < 0.01). In the survival analysis, the hazard ratios of M2BPGi added to each clinical variable were higher than that of each clinical variable alone, and M2BPGi was the only independent prognostic factor for the mortality. **Conclusions:** This study demonstrated that M2BPGi may be a useful biomarker for assessing disease severity and clinical outcomes in hospitalized COVID-19 patients. Combined with conventional clinical assessment, M2BPGi would provide objective and valuable information for prognosis prediction in these critically ill patients. Further studies are warranted to extend its utility in other clinical settings.

## 1. Introduction

The WHO has declared the end of the coronavirus disease 2019 (COVID-19) pandemic by severe acute respiratory syndrome coronavirus-2 (SARS-CoV-2) infection in May 2023 [1,2]. An unexpected increase in COVID-19 cases, however, still continues, and most symptomatic COVID-19 patients present with mild (without pneumonia) and moderate diseases (pneumonia). Some COVID-19 patients may progress to severe (severe pneumonia) and critical diseases (sepsis or septic shock), requiring life-sustaining treatment [3]. It has been reported that hospitalized COVID-19 patients had a 22% and 113% higher risk of developing sepsis and septic shock, respectively, compared with hospitalized patients with influenza [4,5]; about 20% of patients with severe COVID-19 in intensive care units (ICUs) could develop sepsis, and for every hour of delay in appropriate treatment, the survival from septic shock drops by 7.6% [4].

Several clinical variables have been reported to be associated with disease severity and clinical outcomes in COVID-19 [3,6,7,8,9,10,11]. Older age is associated with disease severity and mortality [3,6,7,8,11]. Aging is related with the decreased expression of angiotensin-converting enzyme 2, a functional receptor for the Spike glycoprotein of SARS-CoV-2, which may contribute to poor outcomes of COVID-19 [11]. In addition, severe/critical disease, the sequential organ failure assessment (SOFA) score, and the National Early Warning Score (NEWS) 2 were significantly associated with poor clinical outcomes in COVID-19 [3,8,9,10]. The early detection of COVID-19 patients at risk of severe or critical disease enables timely and effective treatment, and biomarker-based approaches would be more beneficial than clinical variable-based approaches in predicting clinical outcomes in COVID-19 [3,8,9,12,13].

Mac-2 binding protein glycosylation isomer (M2BPGi), a glycoprotein secreted by hepatic stellate cells (HSCs), is a novel biomarker for predicting liver fibrosis (LF) and may reflect liver damage or liver inflammation [14,15,16,17,18]. Liver damage in COVID-19 is defined as COVID-19-related liver injury (LI) regardless of preexisting chronic liver diseases (CLDs); in addition, various medications used for COVID-19 treatment, including antibiotics, antivirals, or antimalarials, could induce LI [19,20]. Early in the COVID-19 pandemic in Wuhan, 78% of 82 non-survivors presented with COVID-19-related LI, even though only 2.4% had preexisting CLDs [21].

To the best of our knowledge, no study has evaluated the prognostic role of M2BPGi in COVID-19. In this study, we aimed to explore the performance of M2BPGi for predicting 30-day mortality and 60-day mortality in hospitalized COVID-19 patients, in comparison with other clinical variables (age, severe/critical disease, SOFA score, and NEWS2). We also investigated whether the addition of M2BPGi to each clinical variable would improve the prognostic value of the metric or render it obsolete.

## 2. Materials and Methods

### 2.1. Study Population

The study population was a total of 53 COVID-19 patients who were admitted to the Yeungnam University Medical Center (YUMC), Daegu, Republic of Korea, during the period from February to June 2020, when the COVID-19 pandemic first surged. The diagnosis of COVID-19 was confirmed by real-time reverse transcription-polymerase chain reaction (RT-PCR) using the Allplex 2019-nCoV assay (Seegene, Seoul, Republic of Korea) with nasopharyngeal swabs or sputum, as described in our previous study [22]. The enrollment criteria were as follows: (1) hospitalized adult patients (aged ≥ 20 years); (2) sufficient residual sera after routine blood testing; and (3) known clinical outcomes (30-day and 60-day mortality) after COVID-19 diagnosis. This study consisted of forward collection of residual left-over samples and retrospective review of medical records. In this study, all the patient data were analyzed anonymously, and neither additional blood sampling nor intervention was performed. The study protocol was approved by the institutional review board of YUMC (approval No. 2020-12-004) before collecting the first sample, and obtaining written informed consent from the study population was exempt. The registry of this study population has been used to generate other data sets that have been reported previously [8,9,22].

Patients’ medical records were reviewed thoroughly to assess demographic, clinical, and laboratory data. At enrollment, disease severity (mild/moderate and severe/critical diseases), SOFA score, and NEWS2 were assessed according to the previous studies [3,23,24]. To assess LF, Fibrosis-4 (FIB-4) was calculated, and FIB-4 ≥ 1.3 was considered intermediate/high risk requiring LF assessment by transient elastography (TE) that was not applicable for these hospitalized COVID-19 patients [25]. Except for one asymptomatic patient, the symptom duration ranged up to 25 days in the remaining 52 symptomatic patients. The duration between COVID-19 diagnosis and admission was heterogeneous; 42 were admitted within 11 days of COVID-19 diagnosis (within the first 48 h [*n* = 30] and between 3 and 11 days [*n* = 12]) and 11 were diagnosed as having COVID-19 after admission (within the first 48 h [*n* = 6], between 3 and 9 days [*n* = 3], and one each on day 20 and 29). Therefore, it was impossible to enroll the study population at the same point in the COVID-19 clinical course. The duration from COVID-19 diagnosis to discharge or death ranged up to 155 days, and the duration from COVID-19 diagnosis to death ranged up to 43 days. Clinical outcomes included 30-day and 60-day mortality, defined as death within 30 and 60 days of COVID-19 diagnosis, respectively. Basic characteristics of the study population are summarized in Table 1. The proportion of patients with mild/moderate and severe/critical diseases was 28.3% (*n* = 15) and 71.7% (*n* = 38]), respectively. There were 33 males, and their median age was 72.0 years. Older patients accounted for 67.9% (*n* = 36) in age ≥ 65 years, 56.6% (*n* = 30) in age ≥ 70 years, and 39.6% (*n* = 21) in age ≥ 75 years. Age and the proportion of older age groups did not differ significantly between mild/moderate and severe/critical disease.

### 2.2. M2BPGi Assay

A total of 239 sera were collected from 53 patients from enrollment to discharge or death; 53 sera were collected at enrollment from 53 patients, and the remaining 186 sera were collected consecutively from 38 patients. Blood samples collected with the BD Vacutainer^®^ SST™ Blood Collection Tube (Becton, Dickinson and Company, Franklin Lakes, NJ, USA) were centrifuged for 10 min at 3000 *g*. The sera were aliquoted and stored at −70 °C until use. Frozen samples were thawed at room temperature and gently mixed immediately before measuring M2BPGi levels. M2BPGi levels were measured using the HISCL M2BPGi assay (Sysmex, Kobe, Japan) with the HISCL-5000 analyzer (Sysmex), which is based on a two-step sandwich chemiluminescent enzyme immunoassay [15,16,17,18]. The M2BPGi level was calculated as follows: cut-off index (COI) = (patient’s serum sample − negative control [NC])/(positive control [PC] − NC). PC was supplied as pre-standardized calibration solution with a 1.0 COI. M2BPGi levels were measured according to the manufacturers’ instructions, and the manufacturer-suggested analytical measuring interval was from 0.1 to 20.0 COI.

### 2.3. Statistical Analysis

Data were presented as number (percentage) or median (interquartile range, IQR). The Shapiro–Wilk test was used to check the normality of data distribution. The Mann–Whitney U test and Wilcoxon signed-rank test were used to compare continuous variables for independent groups and paired groups, respectively. Chi-squared test or Fisher’s exact test were used to compare categorical variables. We compared demographic, clinical, and laboratory data according to disease severity (mild/moderate vs. severe/critical). Four clinical variables (age, severe/critical disease, SOFA score, and NEWS2) and M2BPGi levels at enrollment were compared according to 30-day and 60-day mortality. Older age was divided into three groups: age ≥ 65 years, age ≥ 70 years, and age ≥ 75 years. M2BPGi levels at enrollment were dichotomized using a cut-off of 1.37, which was the optimal cut-off yielding a sensitivity of 100% and specificity from 46.3% to 50.0% for both 30-day and 60-day mortality derived in the receiver operating characteristic (ROC) curve analysis. We also compared the distribution of older age groups and dichotomized M2BPGi levels according to the disease severity and 30-day and 60-day mortality.

We estimated the predictive performance of the single-marker approaches (M2BPGi, age, severe/critical disease, SOFA score, and NEWS2) and the combined approaches (M2BPGi added to each of the other variables) for predicting 30-day and 60-day mortality. The predictive performance was interpreted according to the area under the curve (AUC): <0.5, test not useful; 0.5–0.6, bad; 0.6–0.7, sufficient; 0.7–0.8, good; and 0.9–1.0, excellent [26]. A total of 38 pairs of M2BPGi levels at enrollment (initial) and at discharge or death (follow-up) levels were compared according to the 60-day mortality. We performed a correlation analysis to assess the association between consecutive M2BPGi levels and disease duration from disease onset, based on 60-day mortality. Disease onset was defined as symptom onset or COVID-19 diagnosis. In addition, we analyzed the correlation between M2BPGI levels and clinical variables, and consecutive M2BPGi levels were analyzed along with consecutive vital signs, laboratory data, and FIB-4. The Pearson correlation coefficient (r) was interpreted as follows: 0.0–0.3, negligible; 0.3–0.5, low; 0.5–0.7, moderate; 0.7–0.9, high; and 0.9–1.0, very high [27].

Kaplan–Meier (KM) survival analysis was used to calculate the hazard ratio (HR) with 95% confidence interval (CI) of the single-marker and combined approaches for 30-day mortality and 60-day mortality. The sample size for the KM survival analysis was estimated using log-minus-log transformation, which was suggested for improving the accuracy of the small sample size [28]. The inputs, except for the alternative survival probability, were identical to those mentioned in our previous studies [8,9]. The alternative survival probabilities were set to *S*_1_ (*t*) = 0.226 for 30-day mortality and *S*_1_ (*t*) = 0.283 for 60-day mortality based on the 30-day mortality and 60-day mortality in this study. The sample size for 30-day mortality or 60-day mortality ranged from 9 to 16 with actual power > 0.8 [28]. Accordingly, the sample size of 53 was considered sufficient to perform the KM survival analysis [8,9,22]. No death in the M2BPGi control group (M2BPGi ≤ 1.37 COI) caused the phenomenon of monotone likelihood resulting in the infinite HR of M2BPGi > 1.37 COI for both 30-day and 60-day mortality (Figure S1). We did not report the data based on the previous study, which stated that the infinite HR was not considered reliable [29]. Univariate and multivariate Cox proportional hazard (PH) regression analyses were performed to determine the effect of age, male sex, numbers of comorbidities, disease severity, SOFA score, NEWS2, and M2BPGi on the 30-day mortality and 60-day mortality. MedCalc Software (version 23.0.9, MedCalc Software, Ostend, Belgium) was used for statistical analysis, and *p* < 0.05 was considered statistically significant.

## 3. Results

Table 1 shows the demographic, clinical, and laboratory findings in the study population. The median M2BPGi level (IQR) was 1.9 COI (1.0–3.7) in total patients; M2BPGi levels and the proportion of M2BPGi levels > 1.37 COI differed significantly between mild/moderate and severe/critical diseases (*p* = 0.045 and *p* = 0.022, respectively). However, the FIB-4 and the proportion of FIB-4 ≥ 1.3 did not differ significantly between the two groups. The SOFA score and NEWS2 differed significantly between mild/moderate and severe/critical diseases (both *p* < 0.001).

Table 2 shows the comparison of clinical variables and M2BPGi levels according to the mortality. The age and the proportion of the older age group (age ≥ 70 years and ≥75 years) differed significantly according to the 30-day mortality and 60-day mortality (all *p* < 0.05). The SOFA score, NEWS2, and M2BPGi levels, and the proportion of M2BPGi levels > 1.37 COI differed significantly according to the 30-day and 60-day mortality, except for the proportion of severe/critical disease (all *p* < 0.05).

**Figure 1:**
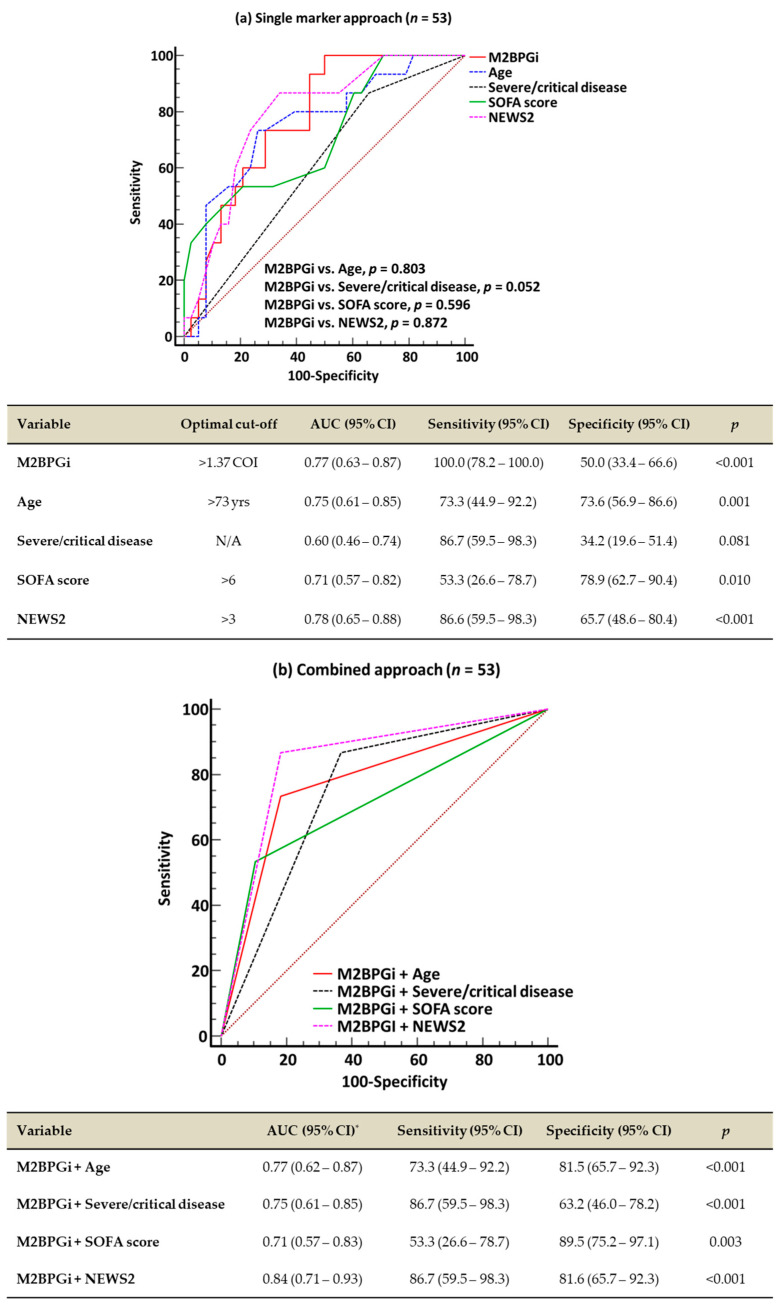

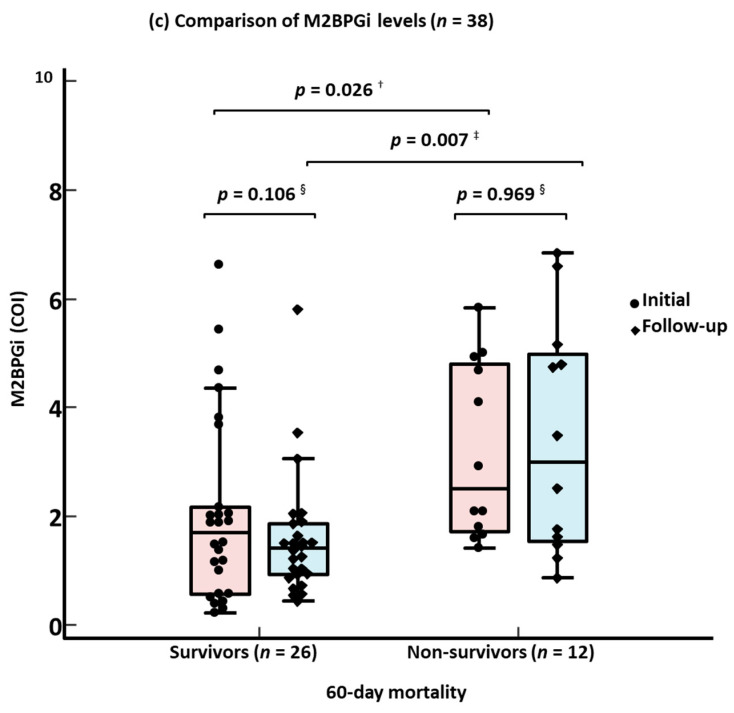
Receiver operating characteristic curve analysis for predicting 60-day mortality and comparison of M2BPGi levels according to 60-day mortality. (**a**) Single-marker approach for 60-day mortality. (**b**) Combined approach for 60-day mortality. (**c**) Comparison of M2BPGi levels according to 60-day mortality. * AUCs of all four combined approaches were comparable. ^†^ Mann–Whitney test for initial M2BPGi levels between survivors and non-survivors. ^‡^ Mann–Whitney test for F/U M2BPGi levels between survivors and non-survivors. ^§^ Wilcoxon signed-rank test between initial and F/U M2BPGi levels. Abbreviations: M2BPGi, Mac-2 binding protein glycosylated isomer; SOFA, sequential organ failure assessment; NEWS2, National Early Warning Score 2; COVID-19, coronavirus disease 2019; yrs, years; AUC, area under the curve; CI, confidence interval; COI, cut-off index; and F/U, follow-up.

In the ROC curve analysis, single-marker approaches using M2BPGi, age, SOFA score, and NEWS2 demonstrated a good performance (AUC 0.71–0.78) for predicting 60-day mortality, except for severe/critical disease (AUC 0.60) (Figure 1a). The combined approaches all demonstrated a good predictive performance (AUC: 0.71–0.84); among them, M2BPGi + NEWS2 showed the highest performance (Figure 1b). The results of the ROC curve analysis for 30-day mortality were similar to the results for 60-day mortality. In the 38 patients with consecutive M2BPGi levels, both the initial and follow-up M2BPGi levels were significantly higher in the non-survivors than in the survivors (survivors vs. non-survivors, 1.7 COI [0.6–2.1] vs. 2.5 COI [1.7–4.8] for initial M2BPGi levels, 1.4 COI [0.9–1.9] vs. 3.0 COI [1.5–4.9] for follow-up M2BPGi levels, all *p* < 0.05) (Figure 1c). The consecutive M2BPGi levels showed a negative correlation with disease duration after symptom onset or COVID-19 diagnosis in survivors (all *p* < 0.001) but not in non-survivors (all *p* > 0.05) (Figure S2). Although the M2BPGi level showed a low correlation with age (r [95% CI], 0.43 [0.18–0.65], *p* = 0.001), it showed negligible correlations with the other variables (−0.2 < r < 0.3).

**Figure 2:**
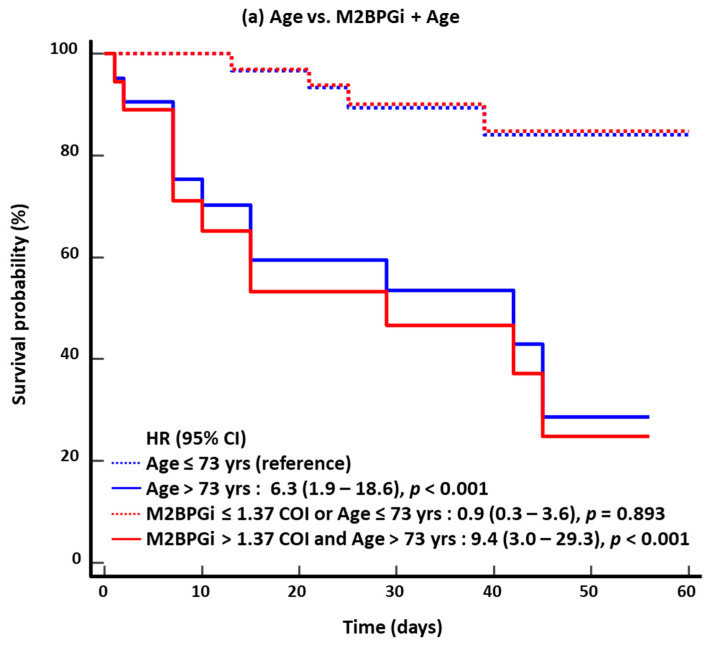

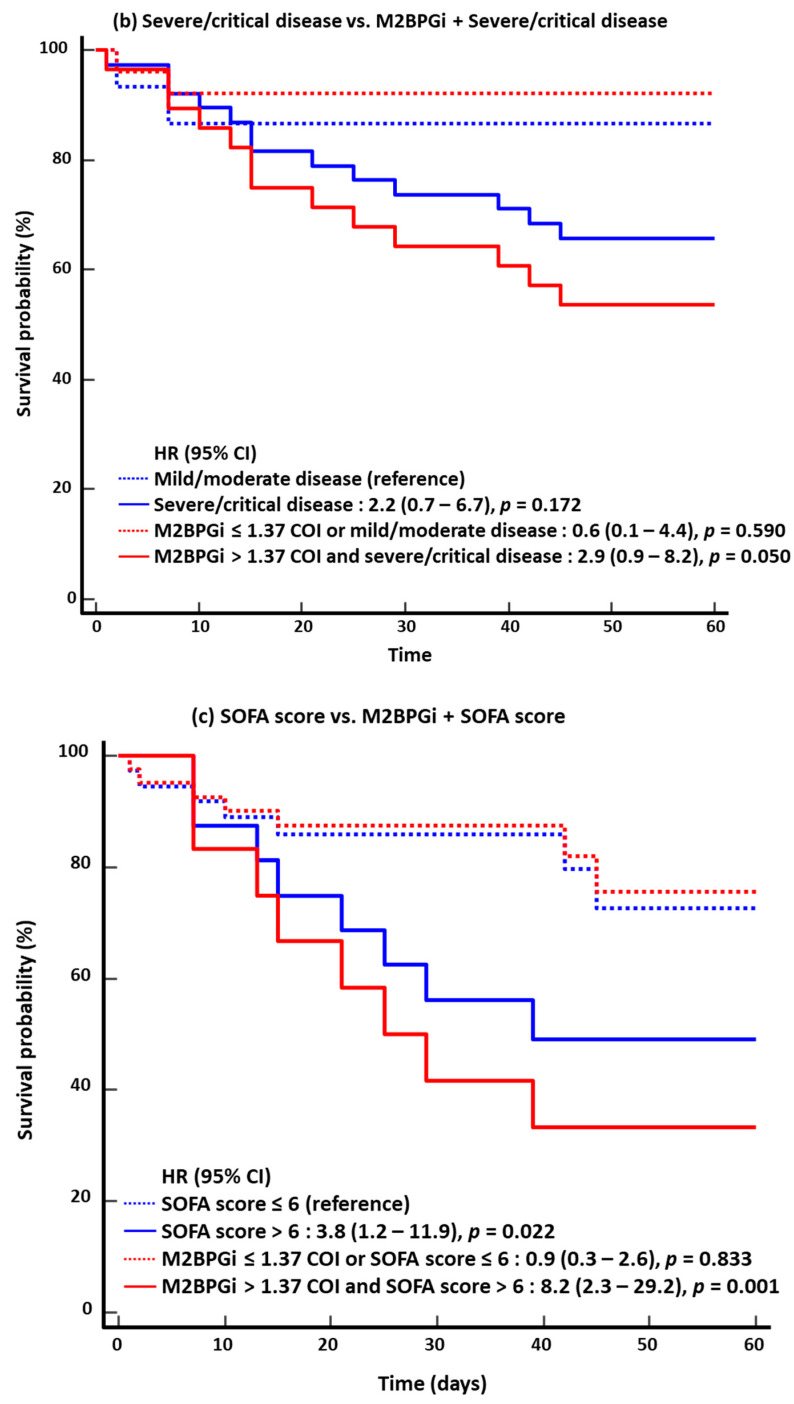

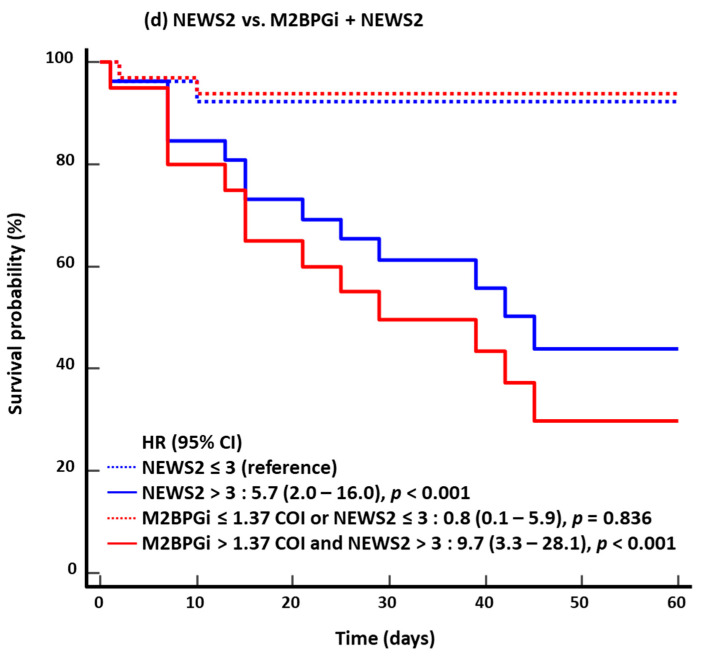
Kaplan–Meier survival analysis for 60-day mortality (*n* = 53). (**a**) Age vs. M2BPGi + Age. (**b**) Severe/critical disease vs. M2BPGi + Severe/critical disease. (**c**) SOFA score vs. M2BPGi + SOFA score. (**d**) NEWS2 vs. M2BPGi + NEWS2. Abbreviations: see Figure 1; HR, hazard ratio.

In the KM survival analysis for 60-day mortality, the HR of the combined approach was higher than that of the single-marker approach: 6.3 for age vs. 9.4 for M2BPGi + age; 2.2 for severe/critical disease vs. 2.9 for M2BPGi + severe/critical disease; 3.8 for SOFA score vs. 8.2 for M2BPGi + SOFA score; and 5.7 for NEWS2 vs. 9.7 for M2BPGi + NEWS2 (Figure 2). The results for 30-day mortality were similar to those for 60-day mortality.

In the univariate Cox PH regression analysis, the age, numbers of comorbidities, SOFA score, NEWS2, and M2BPGi level were significantly associated with both 30-day mortality and 60-day mortality (Table 3). In the multivariate analysis, only the M2BPGi level was significantly associated with 30-day mortality and 60-day mortality (HR = 1.44 and 1.45, respectively, both *p* < 0.05).

## 4. Discussion

This is the first study that investigated the performance of M2BPGi and clinical variables simultaneously for predicting disease severity and clinical outcomes in hospitalized COVID-19 patients. In this study, the majority of the study population (71.7%, *n* = 38) had severe/critical diseases, including 37 patients with critical disease (sepsis or septic shock). Except for age, the M2BPGi level, SOFA score, and NEWS2 were significantly higher in severe/critical disease than in mild/moderate disease.

Our data demonstrated that the M2BPGi levels, age, SOFA score, and NEWS2 were significantly associated with adverse clinical outcomes. Both the single-marker approaches and combined approaches showed good performances for predicting 30-day and 60-day mortality. The AUCs of combined approaches with the addition of M2BPGi were similar to or greater than that of the respective single-marker approach. In particular, the predictive performance of severe/critical disease was changed from bad to good when M2BPGi was added to it. Among the four combined approaches, M2BPGi + NEWS2 showed the greatest performance for predicting 30-day and 60-day mortality. In the KM survival analysis, the HRs of the combined approaches with the addition of M2BPGi were higher than that of respective single-marker approaches. Moreover, M2BPGi was the only independent prognostic factor for 30-day and 60-day mortality. Our data suggests that M2BPGi may be a useful biomarker for predicting clinical outcomes in hospitalized COVID-19 patients.

It is also noteworthy that the M2BPGi level showed a low correlation with age in this study. Old age has been associated with an increased risk of disease severity and poor clinical outcomes in COVID-19 [6,7,8,9]; in our study, among the elderly patients, the proportions of age ≥ 70 years and age ≥ 75 years were significantly higher in the non-survivors than in the survivors. It has also been known that old age is related to increased LF, and the prevalence of significant/advanced LF (F ≥ 2) was the highest at the age ≥ 70 years [30]. Taken together, the combined use of the M2BPGi level and age would help stratify the risks and clinical outcomes more precisely in the COVID-19 patients.

In previous studies, changes in M2BPGi levels reflected the recovery from acute LI and showed a negligible correlation with aspartate aminotransferase (AST) and alanine aminotransferase (ALT), and patients with high M2BPGi levels or FIB-4 showed normal AST or ALT levels [30,31]. Our data also showed that changes in M2BPGi levels reflected the recovery or death from COVID-19, and M2BPGi and LI-related markers showed negligible correlations. In our study, although no patient had preexisting CLD, most of the patients had M2BPGi levels > 1.37 COI and FIB-4 ≥ 1.3, both of which are higher than the respective cut-off for diagnosing LF [14,15,16]. Given that TE was not available for assessing LF, it is possible that some of the COVID-19 patients may have had undetected LF. In a previous study using magnetic resonance elastography, significant/advanced LF (F ≥ 2) was found in approximately 9.5% (*n* = 774) of 8183 subjects who underwent a health check-up [30]. Accordingly, increased M2BPGi levels and FIB-4 may be due to underdiagnosed LF or LF progression triggered by COVID-19-related LI. In contrast to M2BPGi or FIB-4, in most patients the levels of the other LI-related markers (AST, ALT, alkaline phosphatase, gamma-glutamyl transferase, total bilirubin, or direct bilirubin) were within their respective upper reference limits. These findings imply that the M2BPGi level may reflect a different pathophysiology from LI-related markers and would be a more reliable indicator for assessing COVID-19-related LI.

SARS-CoV-2 entry to hosts induces the activation of inflammatory pathways contributing to a cytokine storm, which is characterized by the massive release of proinflammatory cytokines (i.e., interleukin [IL]-1, IL-6, and tumor necrosis factor) and causes multi-organ failure (MOF) in various organs, such as the lungs and liver [19,20,22,32,33,34]. In LI, mediators are released by injured/dying hepatocytes and activated Kupffer cells, and subsequently activate HSCs [35,36]. HSCs transdifferentiate into an activated migratory myofibroblast-like phenotype that expresses smooth muscle alpha-actin, resulting in an increased extracellular matrix, which causes LF [33,35]. Based on our data, M2BPGi levels seem to increase with the progression of inflammation, reflecting the COVID-19 severity.

There are several limitations in this study. First, this was a small-sized and single-center study. Although our sample size was sufficient for the survival analysis and our finding was significant, a larger sample size would be necessary to increase the statistical power and validate significant clinical implications. In addition, further multi-center studies are needed to reflect different clinical scenarios. Second, this study was conducted in an uncontrolled, real-world hospital setting during the first surge of the COVID-19 pandemic; therefore, the heterogeneity of the clinical course of COVID-19 may have affected our data, and our data may not be representative of other variants of SARS-CoV-2. If the study population had a relatively homogeneous clinical course or included other variants of SARS-CoV-2, the data might have been different. Based on this study, further studies using M2BPGi in other infectious diseases or the emergence of other variants of SARS-CoV-2 in a well-controlled hospital setting would be beneficial to ensure the clinical utility of M2BPGi. Third, our patients were mostly elderly patients with critical disease, and the study population did not include a wide range of age and disease severity. This could lead to biased data. A more representative cohort including diverse age groups is needed to ensure the clinical relevance of M2BPGi. Fourth, we focused on the performance of M2BPGi for predicting disease severity and clinical outcomes in hospitalized COVID-19 patients. Due to the lack of information, the prediction of LF in COVID-19 could not be included within the scope of this study. Despite these limitations, this study provided valuable insights into the prognostic role of M2BPGi for predicting disease severity and clinical outcomes in COVID-19.

In conclusion, this is the first study that explored the usefulness of M2BPGi for predicting disease severity and clinical outcomes in hospitalized COVID-19 patients, in comparison with the other clinical variables. M2BPGi was significantly associated with COVID-19 severity and significantly predicted both 30-day and 60-day mortality, comparably to age, the SOFA score, and NEWS2. Severe/critical disease did not significantly predict both 30-day mortality and 60-day mortality, but when M2BPGi was added, it significantly predicted both mortalities. M2BPGi was an independent prognostic factor for both 30-day and 60-day mortality. M2BPGi could be a useful biomarker for predicting disease severity and clinical outcomes in hospitalized COVID-19 patients. Further studies are warranted to implement M2BPGi as a prognostic biomarker for COVID-19 in routine clinical practice as well as to extend its clinical applications in various settings related to critical care.

## Figures and Tables

**Table 1:** Basic characteristics of the study population.

Variable	All Patients (*n* = 53)	Mild/Moderate (*n* = 15) *	Severe/Critical (*n* = 38) *	*p* ^†^
Age (years)	72.0 (62.8–79.0)	67.0 (57.2–75.7)	72.0 (64.0–79.0)	0.098
≥65, *n* (%)	36 (67.9)	8 (53.3)	28 (73.7)	0.156
≥70, *n* (%)	30 (56.6)	6 (40.0)	24 (63.2)	0.129
≥75, *n* (%)	21 (39.6)	4 (26.7)	17 (44.7)	0.350
Male, *n* (%)	33 (62.3)	9 (60.0)	24 (63.2)	0.832
Body mass index (kg/m^2^)	24.1 (22.3–25.8)	22.9 (21.0)	24.3 (22.4–26.1)	0.068
Comorbidities ^‡^, *n* (%)				
*n* = 0	10 (18.9)	3 (20.0)	7 (18.4)	0.486
*n* = 1	12 (22.6)	5 (33.3)	7 (18.4)	
*n* = 2	14 (26.4)	2 (13.4)	12 (31.6)	
*n* ≥ 3	17 (32.1)	5 (33.3)	12 (31.6)	
Hypertension	27 (50.9)	7 (46.7)	20 (52.6)	0.698
Diabetes mellitus	19 (35.8)	3 (20.0)	16 (42.1)	0.204
Malignancy	9 (17.0)	4 (26.7)	5 (13.2)	0.252
Dyslipidemia	9 (17.0)	3 (20.0)	6 (15.8)	0.700
Chronic neurologic conditions	8 (15.4)	3 (20.0)	5 (13.2)	0.672
Chronic heart disease	6 (11.3)	3 (20.0)	3 (7.9)	0.334
Chronic respiratory disease	5 (9.4)	2 (13.4)	3 (7.9)	0.614
Dementia	5 (9.4)	1 (6.7)	4 (10.5)	1.000
Chronic kidney disease	3 (5.7)	0 (0.0)	3 (7.9)	0.549
Connective tissue disease	3 (5.7)	0 (0.0)	3 (7.9)	0.549
Peripheral artery disease	2 (3.8)	1 (6.7)	1 (2.6)	0.789
Symptoms, *n* (%)				
Respiratory symptoms ^§^	35 (66.0)	10 (66.7)	25 (65.8)	0.952
Fever	34 (64.2)	8 (53.3)	26 (68.4)	0.306
General weakness and/or fatigue	23 (43.4)	8 (53.3)	15 (39.5)	0.363
Gastrointestinal symptoms ^‖^	9 (17.0)	3 (20.0)	6 (15.8)	0.700
Neurological symptoms ^¶^	6 (11.3)	1 (6.7)	5 (13.2)	0.662
Symptom duration (day)	4.0 (1.0–7.5)	7.0 (5.0–16.0)	1.5 (0.0–7.0)	<0.001
COVID-19 dx to admission, *n* (%)				
COVID-19 dx + ≤ 48 h	30 (56.6)	6 (40.0)	24 (63.2)	0.090
COVID-19 dx + 3 to 11 days	12 (22.6)	3 (20.0)	9 (23.7)	
COVID-19 dx after admission **	11 (20.8)	6 (40.0)	5 (13.2)	
COVID-19 dx to enrollment (day)	3.0 (0.0–11.0)	3.0 (0.2–37.5)	3.0 (0.0–9.0)	0.189
Hospital stays (day)	29.0 (20.8–49.0)	45.0 (17.3–58.8)	27.5 (21.0–42.0)	0.458
Vital signs				
Systolic BP (mm Hg)	120.0 (110.0–141.3)	120.0 (110.0–138.0)	127.5 (110.0–147.0)	0.352
Diastolic BP (mm Hg)	72.0 (70.0–80.0)	70.0 (68.5–78.5)	74.5 (70.0–80.0)	0.523
Pulse rate (beats/min)	82.0 (74.0–95.5)	81.0 (71.5–89.0)	83.0 (77.0–98.0)	0.195
Respiration rate (breaths/min)	20.0 (20.0–23.0)	20.0 (19.0–20.0)	20.5 (20.0–24.8)	0.022
Body temperature (°C)	37.1 (36.8–37.7)	36.8 (36.6–37.1)	37.3 (36.9–37.8)	0.033
Oxygen saturation (%)	96.0 (94.4–97.0)	97.0 (96.0–97.0)	95.5 (93.6–97.0)	0.148
Laboratory data				
White blood cells (×10^9^/L)	6.1 (4.8–8.9)	4.8 (4.3–5.9)	6.8 (5.2–9.8)	0.029
Neutrophils (×10^9^/L)	4.3 (3.1–6.7)	3.2 (2.5–3.6)	5.2 (3.7–7.7)	0.004
Lymphocytes (×10^9^/L)	1.1 (0.6–1.5)	1.5 (1.2–1.8)	0.8 (0.6–1.2)	<0.001
Aspartate aminotransferas (U/L)	30.0 (23.0–43.5)	30.0 (23.7–41.7)	30.0 (23.0–43.0)	0.751
Alanine aminotransferase (U/L)	24.0 (14.8–37.3)	23.0 (11.5–37.7)	24.5 (15.0–35.0)	0.607
Alkaline phosphatase (U/L)	72.0 (63.0–86.0)	70.0 (63.0–74.0)	73.0 (63.0–87.0)	0.326
γ-glutamyl transferase (U/L)	28.0 (20.2–48.0)	24.0 (18.0–29.5)	30.5 (23.0–54.5)	0.062
Lactate dehydrogenase (U/L)	531.0 (420.2–750.0)	418.0 (338.0–591.0)	577.0 (449.7–827.3)	0.017
Total bilirubin (umol/L)	12.5 (9.5–18.2)	15.1 (9.7–17.6)	11.9 (9.4–25.8)	0.843
Direct bilirubin (umol/L)	3.5 (2.2–6.7)	2.9 (1.5–4.7)	4.0 (2.4–11.3)	0.113
Creatinine (umol/L)	76.9 (57.0–95.4)	60.1 (55.0–89.9)	80.0 (63.6–110.5)	0.141
Lactate (mmol/L)	1.8 (1.2–2.3)	1.6 (1.0–2.0)	1.8 (1.3–2.5)	0.194
C-reactive protein (mg/L)	39.1 (4.7–132.3)	4.0 (0.4–38.9)	63.8 (19.6–151.5)	0.003
M2BPGi (COI)	1.9 (1.0–3.7)	0.8 (0.4–2.6)	2.0 (1.4–3.8)	0.045
>1.37 ^††^, *n* (%)	34 (64.2)	6 (40.0)	28 (73.7)	0.022
Liver fibrosis score				
FIB-4	2.2 (1.2–4.7)	1.9 (1.5–2.4)	2.6 (1.2–4.3)	0.323
≥1.3 ^‡‡^	39 (73.6)	12 (80.0)	27 (71.1)	0.509
Severity assessment				
SOFA score	4.0 (1.0–7.0)	0.0 (0.0–1.0)	5.0 (3.0–7.0)	<0.001
NEWS2	3.0 (2.0–6.0)	2.0 (0.0–3.0)	5.0 (3.0–8.0)	<0.001
Sepsis/septic shock	31 (58.5)/6 (11.3)	0 (0.0)/0 (0.0)	31 (81.6)/6 (15.8)	NA
Treatment, *n* (%)				
Supplemental oxygen therapy	23 (43.4)	2 (13.3)	21 (55.3)	0.006
Antibiotics	34 (64.2)	10 (66.7)	24 (63.2)	0.812
Azithromycin	29 (54.7)	8 (53.3)	21 (55.3)	0.899
3rd-generation cephalosporins	20 (37.7)	6 (40.0)	14 (36.8)	0.832
Piperacillin/tazobactam	2 (3.8)	0 (0.0)	2 (5.3)	1.000
Fluroquinolone	2 (3.8)	0 (0.0)	2 (5.3)	1.000
Lopinavir/ritonavir	26 (49.1)	10 (66.7)	16 (42.1)	0.110
Hydroxychloroquine	22 (41.5)	7 (46.7)	15 (39.5)	0.635
Clinical outcomes, *n* (%)				
ICU admission	14 (26.4)	1 (6.7)	13 (34.2)	0.080
Ventilator use	12 (22.6)	0 (0.0)	12 (31.6)	0.012
ECMO use	7 (13.2)	0 (0.0)	7 (18.4)	0.171
30-day mortality ^§§^	12 (22.6)	2 (13.3)	10 (26.3)	0.471
60-day mortality ^§§^	15 (28.3)	2 (13.3)	13 (34.2)	0.182

Data are represented as number (percentage) or median (interquartile range). * Mild disease (*n* = 1), moderate disease (*n* = 14), severe disease (*n* = 1), and critical disease (*n* = 37). ^†^ *p* values were calculated between non-critical and critical disease patients. ^‡^ No patients had preexisting chronic liver diseases. ^§^ Respiratory symptoms included dyspnea, cough, sputum, hemoptysis, chest pain, rhinorrhea, nasal obstruction, and sore throat. ^‖^ Gastrointestinal symptoms included anorexia, abdominal distension, abdominal pain, and diarrhea. ^¶^ Neurological symptoms included headache, dizziness, mental change, motor weakness, and drooling. ** Of the 53 COVID-19 patients, 11 were diagnosed as having COVID-19 after admission (within the first 48 h [*n* = 6], between 3 and 9 days [*n* = 3], and one each on day 20 and 29). ^††^ The optimal cut-off value of M2BPGi for both 30-day mortality and 60-day mortality was 1.37 COI in the receiver operating characteristic curve analysis. ^‡‡^ FIB-4 cut-off of 1.3 was regarded as an intermediate/high risk requiring liver fibrosis assessment by transient elastography [25]. ^§§^ 30-day mortality and 60-day mortality indicate mortality within 30 days and 60 days of the diagnosis of COVID-19, respectively. Abbreviations: COVID-19, coronavirus disease 2019; dx, diagnosis; BP, blood pressure; M2BPGi, Mac-2 binding protein glycosylated isomer; COI; cut-off index; FIB-4, fibrosis-4; SOFA, sequential organ failure assessment; NEWS2, National Early Warning Score 2; NA, not available; ICU, intensive care unit; ECMO, extracorporeal membrane oxygenation.

**Table 2:** Comparison of clinical variables and M2BPGi level according to 30-day mortality and 60-day mortality.

Variable	30-Day Mortality			60-Day Mortality		
	Survivors (*n* = 41)	Non-Survivors (*n* = 12)	*p*	Survivors (*n* = 38)	Non-Survivors (*n* = 15)	*p*
Age (years)	69.0 (61.7–77.0)	78.5 (73.5–83.0)	0.016	68.5 (61.0–75.0)	79.0 (72.7–83.5)	0.004
≥65, *n* (%)	26 (63.4)	10 (83.3)	0.296	23 (60.5)	13 (86.7)	0.102
≥70, *n* (%)	20 (48.8)	10 (83.3)	0.047	18 (47.4)	12 (80.0)	0.036
≥75, *n* (%)	12 (29.3)	9 (75.0)	0.007	10 (26.3)	11 (73.3)	0.001
Severe/critical disease, *n* (%)	28 (68.3)	10 (83.3)	0.471	25 (65.8)	13 (86.7)	0.182
SOFA score	4.0 (0.0–5.2)	7.5 (3.0–9.5)	0.018	3.5 (0.0–5.0)	7.0 (3.0–9.0)	0.016
NEWS2	3.0 (1.0–5.3)	5.5 (4.0–9.0)	0.010	3.0 (1.0–4.0)	6.0 (4.2–9.0)	0.001
M2BPGi (COI)	1.5 (0.5–2.4)	2.7 (1.8–4.7)	0.011	1.4 (0.5–2.2)	2.9 (1.8–4.8)	0.002
>1.37, *n* (%)	22 (53.7)	12 (100.0)	0.002	19 (50.0)	15 (100.0)	<0.001

Data are represented as number (percentage) or median (interquartile range). *p* values were calculated using Mann–Whitney U test for continuous variables and chi-squared test (or Fisher’s exact test) for categorical variables. Abbreviations: SOFA, sequential organ failure assessment; NEWS2, National Early Warning Score 2; M2BPGi, Mac-2 binding protein glycosylated isomer; and COI; cut-off index.

**Table 3:** Cox proportional hazard regression analysis for predicting 30-day and 60-day mortality.

Variable	Univariate		Multivariate	
	**HR (95% CI)**	***p***	**HR (95% CI)**	***p***
***30-day mortality***				
Age	1.06 (1.01–1.12)	0.031	1.01 (0.93–1.10)	0.753
Male	0.59 (0.19–1.83)	0.364		
Comorbidities (*n*)	1.71 (1.04–2.82)	0.033	1.69 (0.89–3.23)	0.111
Disease severity	1.47 (0.69–3.12)	0.319		
SOFA score	1.24 (1.03–1.48)	0.018	1.17 (0.63–1.47)	0.190
NEWS2	1.16 (1.03–1.31)	0.013	1.09 (0.92–1.28)	0.317
M2BPGi	1.27 (1.01–1.62)	0.048	1.44 (1.05–1.98)	0.025
***60-day mortality***				
Age	1.07 (1.02–1.12)	0.010	1.03 (0.96–1.11)	0.370
Male	0.65 (0.24–1.80)	0.412		
Comorbidities (*n*)	1.55 (1.01–2.39)	0.048	1.46 (0.86–2.49)	0.159
Disease severity	1.71 (0.81–3.59)	0.159		
SOFA score	1.23 (1.05–1.45)	0.012	1.12 (0.90–1.38)	0.300
NEWS2	1.18 (1.06–1.32)	0.002	1.15 (0.98–1.34)	0.070
M2BPGi	1.30 (1.06–1.60)	0.012	1.45 (1.09–1.92)	0.010

Abbreviations: HR, hazard ratio; CI, confidence interval; *n*, number; SOFA, sequential organ failure assessment; NEWS2, National Early Warning Score 2; and M2BPGi, Mac-2 binding protein glycosylated isomer.

## Data Availability

The data presented in this study are available from the corresponding author on reasonable request.

## Supplementary Materials

The supporting information can be downloaded at https://www.mdpi.com/article/10.3390/diagnostics15070937/s1. Figure S1. Kaplan-Meier survival analysis of M2BPGi for 30-day mortality and 60-day mortality (*n* = 53). Due to the phenomenon of monotone likelihood caused by no death in M2BPGi control group (M2BPGi ≤ 1.37 COI, the HR of M2BPGi was infinite for both 30-day mortality and 60-day mortality. (a) 30-day mortality. (b) 60-day mortality. Abbreviations: M2BPGi, Mac-2 binding protein glycosylated isomer; COI, cut-off index; HR, hazard ratio. Figure S2. Correlation between consecutive M2BPGi levels and disease duration according to 60-day mortality (*n* = 239). (a) Consecutive M2BPGi levels according to disease duration since symptom onset. (b) Consecutive M2BPGi levels according to disease duration since COVID-19 diagnosis. Abbreviations: M2BPGi, Mac-2 binding protein glycosylated isomer; COI, cut-off index; COVID-19, coronavirus disease 2019; CI, confidence interval.
