# Supplementary material for: Novel Usefulness of M2BPGi for Predicting Severity and Clinical Outcomes in Hospitalized COVID-19 Patients
Source: Diagnostics (Basel). 2025 Apr 6;15(7):937. doi: 10.3390/diagnostics15070937 (PMC11989196; doi:10.3390/diagnostics15070937)
Supplement: Supplementary file 1 [file diagnostics-15-00937-s001.zip › diagnostics-3578584-supplementary.pdf]

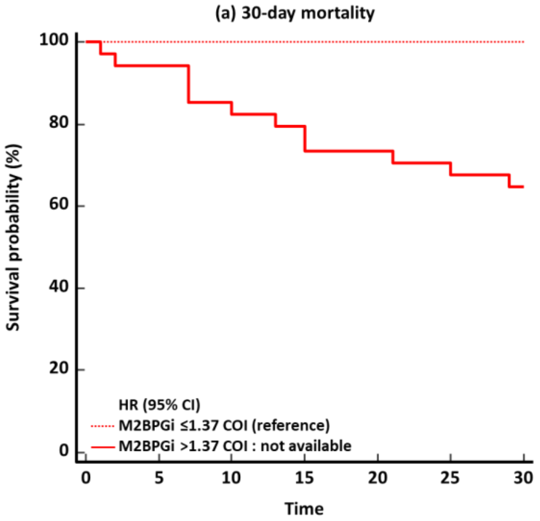


Number at risk

| | | | | | | | |
|---|---|---|---|---|---|---|---|
| M2BPGi ≤1.37 COI | | | | | | | |
| | 19 | 19 | 19 | 19 | 19 | 19 | 0 |
| M2BPGi >1.37 COI | | | | | | | |
| | 34 | 28 | 25 | 22 | 21 | 19 | 0 |

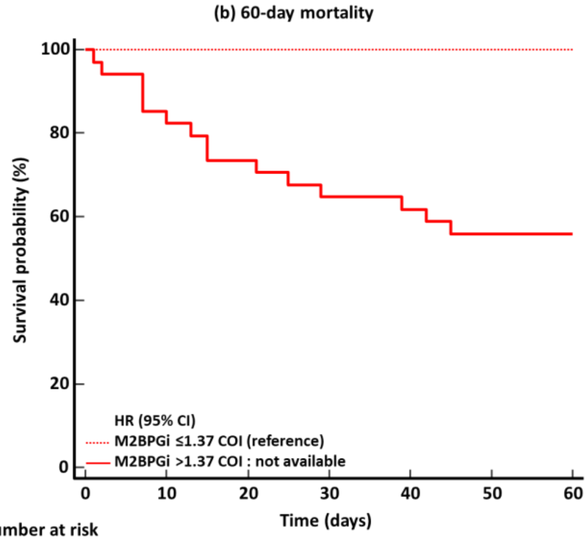


Number at risk

| | | | | | | | |
|---|---|---|---|---|---|---|---|
| M2BPGi ≤1.37 COI | | | | | | | |
| | 19 | 19 | 19 | 19 | 19 | 19 | 0 |
| M2BPGi >1.37 COI | | | | | | | |
| | 34 | 28 | 25 | 22 | 21 | 19 | 0 |

**Figure S1.** Kaplan-Meier survival analysis of M2BPGi for 30-day mortality and 60-day mortality ($n$ = 53). Due to the phenomenon of monotone likelihood caused by no death in M2BPGi control group (M2BPGi ≤ 1.37 COI, the HR of M2BPGi was infinite for both 30-day mortality and 60-day mortality. (a) 30-day mortality. (b) 60-day mortality. Abbreviations: M2BPGi, Mac-2 binding protein glycosylated isomer; COI, cut-off index; HR, hazard ratio.

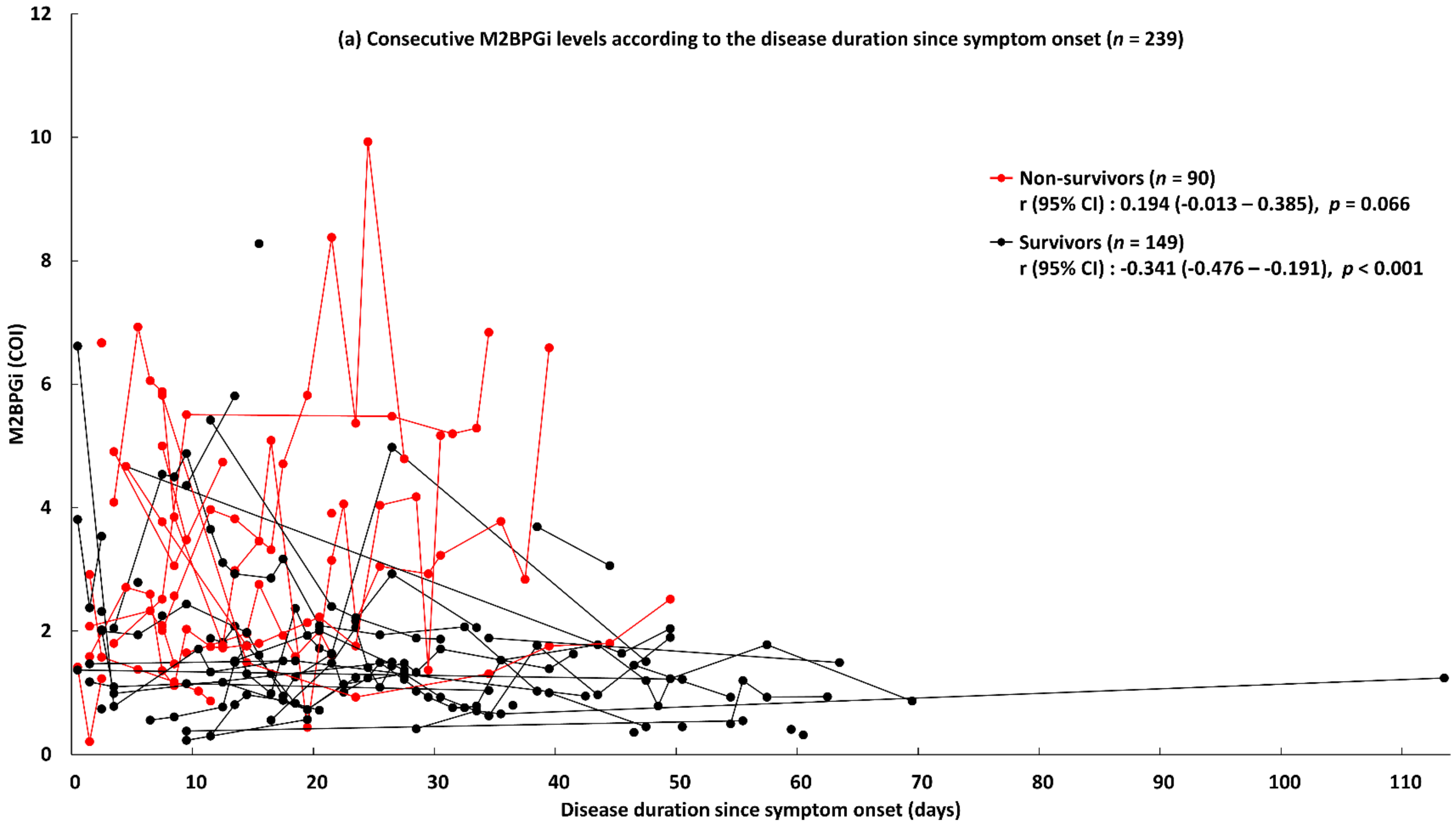

(a) Consecutive M2BPGi levels according to the disease duration since symptom onset (n = 239)
M2BPGi (COI)
12
10
8
6
4
2
0
0
10
20
30
40
50
60
70
80
90
100
110
Disease duration since symptom onset (days)
Non-survivors (n = 90)
r (95% CI) : 0.194 (-0.013 – 0.385), p = 0.066
Survivors (n = 149)
r (95% CI) : -0.341 (-0.476 – -0.191), p < 0.001

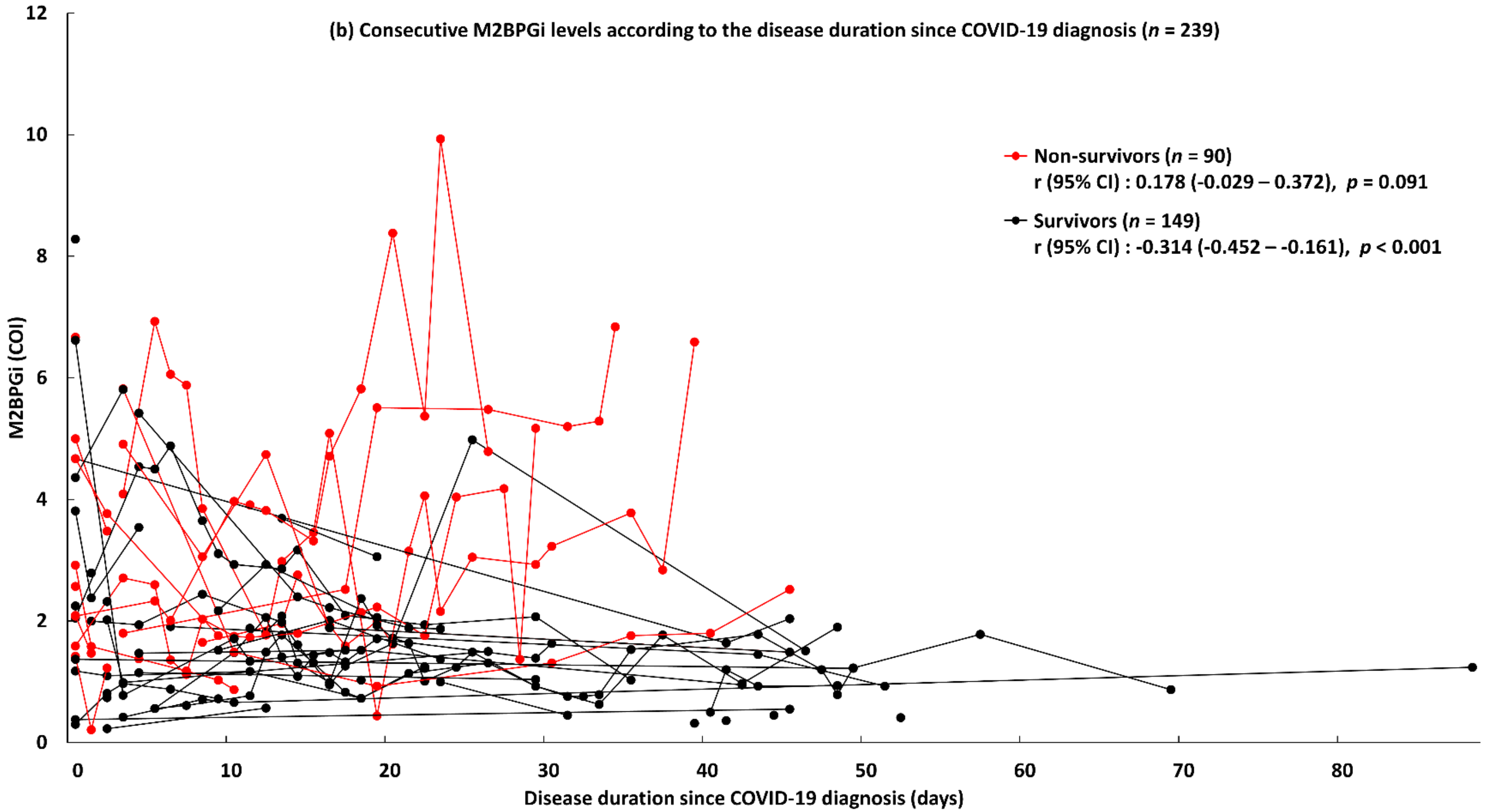


**Figure S2.** Correlation between consecutive M2BPGi levels and disease duration according to 60-day mortality (*n* = 239) (a) Consecutive M2BPGi levels according to disease duration since symptom onset. (b) Consecutive M2BPGi levels according to disease duration since COVID-19 diagnosis. Abbreviations: M2BPGi, Mac-2 binding protein glycosylated isomer; COI, cut-off index; COVID-19, coronavirus disease 2019; CI, confidence interval.
